# Case Report: Eosinophilic fasciitis with PM-Scl antibody positivity: expanding the serological spectrum

**DOI:** 10.3389/fmed.2026.1870461

**Published:** 2026-07-27

**Authors:** Alvina Zanib, Brandon Manicom, Anna O'Rourke, Kieran Murray

**Affiliations:** 1 School of Medicine, University of Limerick, Limerick, Ireland; 2 Infectious Disease Department, Health Service Executive Mid West, Limerick, Ireland; 3 Rheumatology Department, Health Service Executive Mid West, Limerick, Ireland

**Keywords:** autoimmune overlap, eosinophilic fasciitis, fascial inflammation, inflammatory myopathy, PM-Scl antibody, scleroderma

## Abstract

Eosinophilic fasciitis (EF) is a rare connective tissue disorder characterized by symmetrical or asymmetrical skin induration, fascial inflammation, and peripheral eosinophilia. We report the case of a middle-aged man presenting with left knee pain and swelling, progressive skin induration and pigmentation changes on the thigh and trunk. Blood workup showed peripheral eosinophilia and weakly positive PM-Scl-100 antibodies. MRI demonstrated fascial thickening with subcutaneous edema and fluid tracking along fascial planes, as well as a moderate knee effusion. Muscle biopsy confirmed eosinophilic fasciitis without muscle involvement. He was treated with high-dose prednisolone, methotrexate as a steroid-sparing agent, and adjunctive bone and infection prophylaxis, with marked clinical improvement at follow-up.

This case highlights the diagnostic challenges in atypical presentation of eosinophilic fasciitis in the presence of overlap autoantibodies. Careful clinicopathological correlation is critical to exclude other differentials as EF can mimic systemic sclerosis or myositis. Biopsy can improve diagnostic certainty particularly when encountering auto-antibodies that have some association with other diagnoses.

## Introduction

Eosinophilic fasciitis (EF), first described by Shulman (1), is a rare connective tissue disorder characterized by inflammation and fibrosis of the fascia, often accompanied by peripheral eosinophilia and skin induration with a distinctive “peau d'orange” appearance or groove sign (linear depressions along superficial veins, with increasing prominence on limb elevation) (2). The condition typically affects the extremities and trunk and may follow strenuous exercise or trauma.

Articular manifestations are recognized in EF, with non-erosive arthritis and joint effusions reported in up to 40% of patients (4). Because EF is rare and overlaps clinically with other sclerosing or eosinophilic disorders, timely recognition remains challenging. With limited EF cases reported in the literature to date, case reports play a crucial role in expanding awareness of its heterogeneity.

We present the case of a 38-year-old man with oligoarthritis, skin thickening and pigmentation with a weak positive anti-PM-SCL 100 autoantibody test. His Biopsy confirmed EF, highlighting the importance of considering EF in patients presenting with skin tightening and arthritis.

## Patient information

A 38-year-old man without medical history and or any regular medications, presented with a 7-month history of left leg pain and left knee swelling and stiffness, associated with skin tightening. His stiffness and pain later progressed to involve the left ankle, both wrists, and the lower back. The patient noted a significant restriction of movement and a feeling of tightness around his left knee. He also complained about skin discoloration in form of non-itchy patches on his trunk and left thigh along with hair loss. He denied fever or weight loss. There were no symptoms of progressive shortness of breath. There was no history of trauma or infections preceding his illness. There was no significant travel history. He had no history of viral illness or sexually transmitted infection. His family history was notable for an aunt with rheumatoid arthritis complicated with Felty's syndrome and an uncle with inclusion body myositis. The patient worked as a welder. He never smoked and reported drinking 3 standard drinks of alcohol per week. He denied any illicit drug use.

On examination he had small left knee and ankle effusions. The left leg showed patchy hyper- and hypopigmented rash along with skin tethering and induration, especially over the lateral thigh and calf (Figure 1). His right lower trunk had patchy hypopigmented areas over the flank and iliac crest region with skin thickening (Figure 2). There were no further rashes, No Gottron papules and no heliotrope rash were noted. There wasn't any pitting edema, and typical groove sign was also not seen. He had no oral ulceration. Movement on the left knee was limited but power was 5/5. No weakness of proximal muscles was noted.

**Figure 1:**
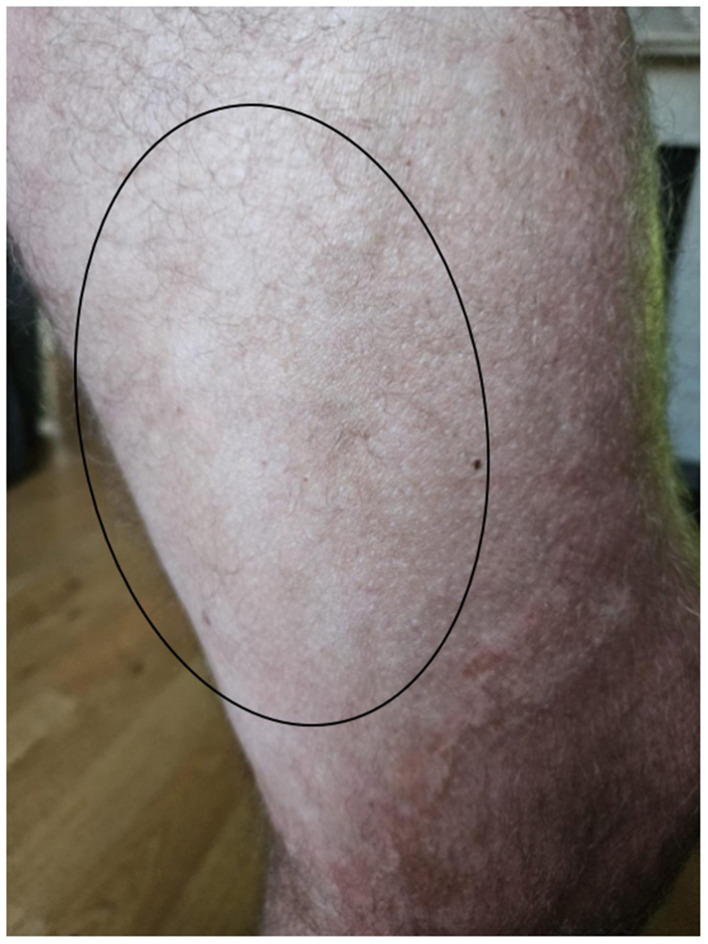
The left leg shows patchy hyperpigmentation and hypopigmentation, along with a healing surgical biopsy site. There is skin tethering and possible subcutaneous induration, especially over the lateral thigh and calf—typical of EF. No clear pitting oedema or joint swelling visible.

**Figure 2:**
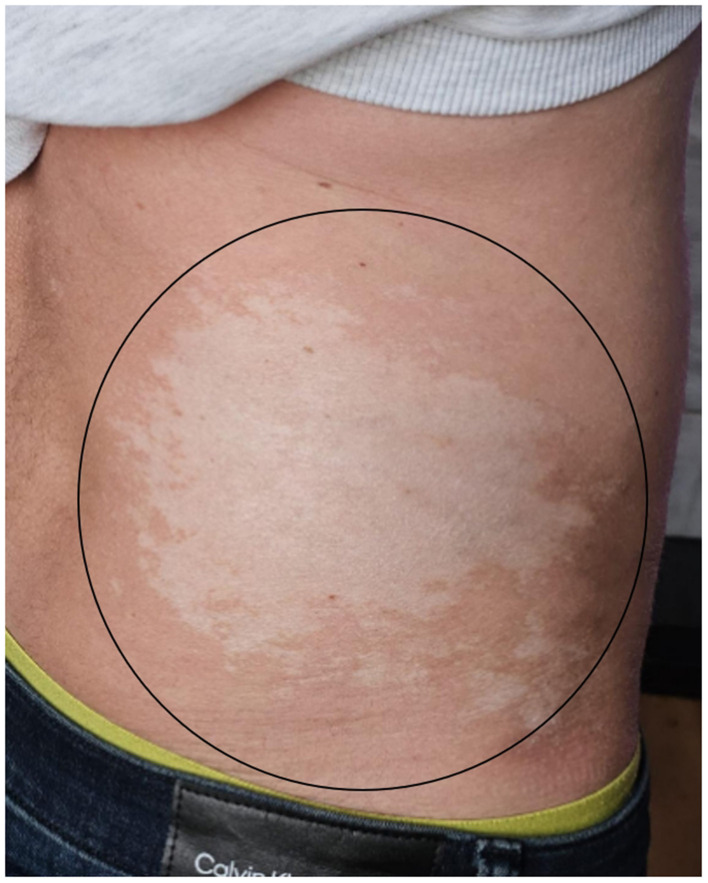
Patchy hypopigmented areas over the flank and iliac crest region.

## Diagnostic assessment

Initial blood tests (Table 1) were notable for peripheral eosinophilia and raised Immunoglobulin A (IgA) and G (IgG) levels. IgG subclass analysis showed mild increases in both IgG1 and IgG3, with normal IgG2 and IgG4. He had a weakly positive anti-PM/SCL-100 antibody. ANCA, ANA, anti-CCP, RF, Mi-2 Alpha, Mi-2 Beta, TIF1-Gamma, MDA5, NXP2, SAE1, KU, PM-SCL-75, Jo-1, SRP, Pl-7, Pl-12, EJ, OJ and RO-52 antibodies were all negative. Antibodies were tested at an accredited laboratory using qualitative analysis using a Immunoblot/Euroblot One Analyser. A weakly positive result suggests that there was some reactivity on the assay, but not all samples reacted.

**Table 1:** Pertinent laboratory data at baseline and at follow up.

Blood test	Baseline	6 week follow up	Normal ranges (units)
White blood cell	7.98	8.09	4–11 × 10^9^/L
Hemoglobin	15.1	16.3	13.5–16.5 g/dL
Platelet	235	258	150–450 × 10^9^/L
Neutrophils	4.43	**7.22**	2–7 × 10^9^/L
Lymphocytes	1.38	0.61	1–3 × 10^9^/L
Monocytes	0.72	0.24	0.2–1 × 10^9^/L
Eosinophils	**1.37**	**0.00**	0.02–0.5 × 10^9^/L
CRP	5	1	< 5 mg/L
ESR	8	2	< 20 mm/hr
Immunoglobulin-G	**17.5**	11.5	6.0–16.0 g/L
Immunoglobulin-A	**4.7**	4.0	0.70–4.00 g/L
Immunoglobulin-M	0.9	0.8	0.4–2.38 g/L
C3	1.4	1.3	0.9–1.8 g/L
C4	0.37	0.27	0.10–0.40 g/L
Creatine kinase	65	**354**	40–320 U/L

Bold values indicate Eosinophils elevated at baseline with increased igG and igA values returning to baseline at 6 week follow up however mildly raised creatine kinase noted on 6 week follow-up.

Chest Radiography was unremarkable. Magnetic resonance imaging of left leg showed diffuse subcutaneous oedema involving left lower limb along with fascial enhancement and fluid tracking along the fascial planes. Diffuse oedema was noted around the right knee (Figures 3, 4B). Partially visualized distal sartorius demonstrated diffuse oedema raising possibility of myositis. In addition, moderate knee effusion was noted (Figure 4A).

**Figure 3:**
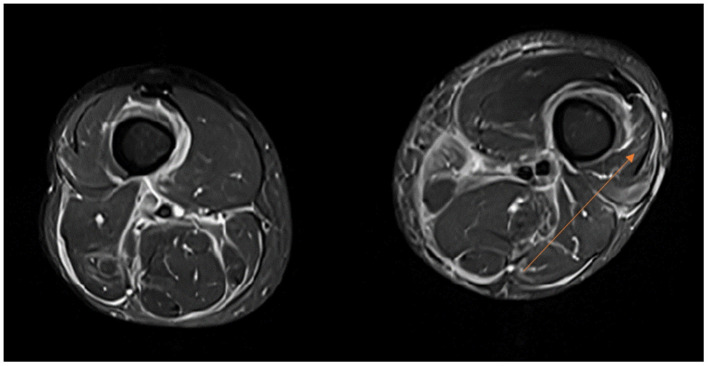
Magnetic resonance imaging of left leg showed diffuse sartorius oedema and extensive subcutaneous oedema with fluid tracking along the fascial planes.

**Figure 4:**
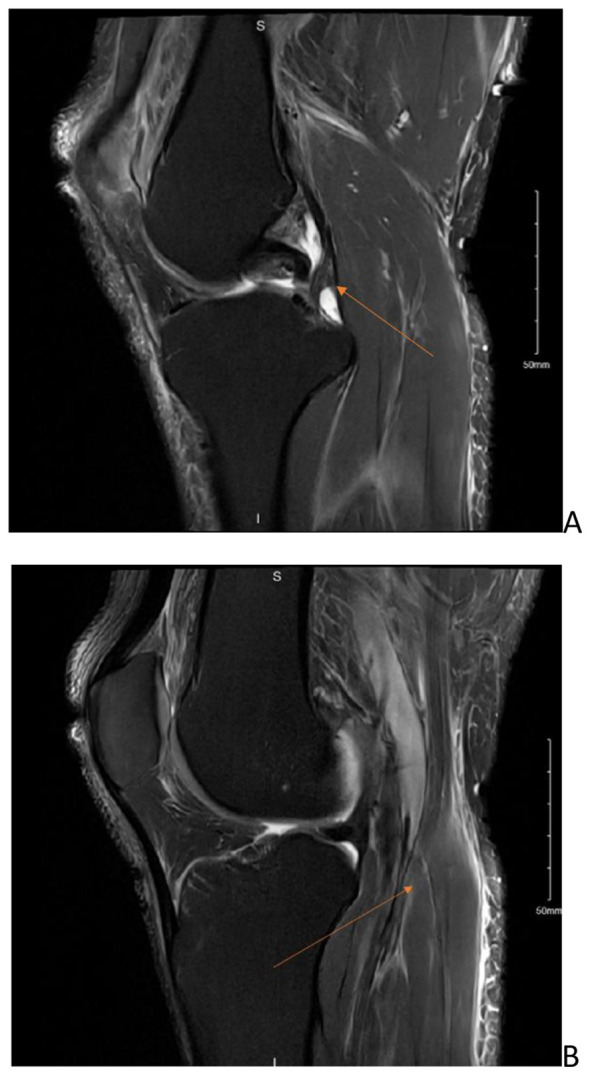
Magnetic Resonance Imaging T2 Weighted sequence showing **(A)** Knee effusion and **(B)** Muscle edema.

Deep fascia and muscle biopsy of the left recto-femoris muscle showed chronic inflammation with prominent eosinophilic and lymphocytic infiltrates in the perimysium and fascia extending into the subperimysial endomysium (Figures 5A, B). The perimysial collagen appears fragmented, with no evidence of inflammation within the myofibres, arguing against a diagnosis of myositis. The muscle biopsy with the associated peripheral eosinophilia and hypergammaglobulinemia suggested a diagnosis of Eosinophilic Fasciitis.

**Figure 5:**
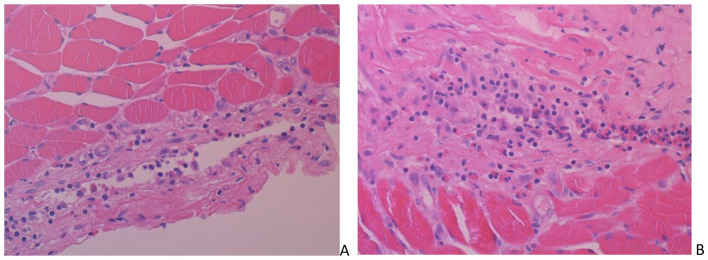
A: Inflammation centered on the perimysium **(A)** and fascia **(B)** with frequent eosinophils and fragmentation of the collagen. Inflammation extends into the superficial endomysium Myofibre inflammation is not present.

## Therapeutic intervention

Following confirmation of the diagnosis, the patient was commenced on oral prednisolone 60 mg daily along with calcium, vitamin D and prophylactic co-trimoxazole with plan to follow up at 6 weeks' time.

## Follow-up and outcomes

At his 6-week outpatient follow up, the patient reported approximately 80% clinical improvement, with a marked reduction in pain, swelling, and skin tightness of the lower limbs.

His fascial biopsy site was healed well. Blood results showed significant improvement with normalization of eosinophilia (Table 1). The CK was moderately raised however it was associated with clinical improvement of patient symptoms

In view of the good initial response, a prednisolone tapering regimen was initiated, and methotrexate was introduced as a steroid-sparing agent along with folic acid supplementation. Arrangements were made for follow up CRP, ESR, Urea and Electrolyte and Full Blood Count in the community, and the patient was scheduled for rheumatology clinic reassessment in 3 months' time.

## Discussion

Eosinophilic fasciitis (Shulman's disease) is a rare connective tissue disorder characterized by inflammation and fibrosis of the fascia, leading to skin induration, tightening, and functional impairment (1, 7). It was first described in 1974 but remains underdiagnosed due to its overlap with scleroderma-spectrum disorders (7).

EF is mostly considered idiopathic but several associations has been seen in literature, including, strenuous exercise (8), medications (including statins, ramipril, heparin, immune check point inhibitors (including pembrolizumab), and anti-TNF agents) (9–13); hematological discorders (leukemia, lymphoma, aplastic anemia), cancer (lung, melanoma), haemodialysis (14–16), Lyme disease (17), and graft-vs.-host disease (18). There was no specific association found in our patient.

Pathogenesis of EF is unclear but likely involves an autoimmune mechanism given clinical features, laboratory results and good response to steroids (19, 20). As seen in literature, dysregulated activation of eosinophils, fibroblast and Th lymphocytes leads to productions of cytokines (notably IL-5, IL-2, TGF- β, interferon-γ) causing excessive production of fibronectin and type I collagen, leading to thickening of skin and fascia. (19, 21).

Laboratory findings in eosinophilic fasciitis commonly include transient peripheral eosinophilia (20–23), which does not necessarily correlate with disease severity. Patients may also show elevated inflammatory markers such as mild to moderate elevation of erythrocyte sedimentation rate (ESR) and C-reactive protein (CRP), along with polyclonal hypergammaglobulinemia (19, 24, 25). In such cases, immunoelectrophoresis is useful to exclude underlying hematological disorders which may trigger EF.

Specific antibodies are usually absent in EF (25, 26). Antinuclear antibodies and rheumatoid factor were identified in 10% of EF patients (3). Anti-Ha antibodies have been reported in one patient with eosinophilic fasciitis, suggesting a potential overlap or association with anti-synthetase syndrome or other forms of myositis (27, 28). Isolated weak Anti-PM/Scl antibodies were identified in our patient. Lack of proximal muscle weakness on examination and history and no other specific skin findings that are not better explained by eosinophilic fasciitis along with a lack of symptoms suggestive of interstitial lung disease make a weak anti-PM/Scl finding less clinically concerning for a myositis overlap syndrome. Available literature suggests that weakly positive anti-PM/Scl 100 antibodies may require monitoring as these patients may develop connective tissue diseases over time (5, 6). No previous cases of EF from literature have reported positive anti-PM/scl antibodies.

The patient had a mildly elevated Creatinine Kinase (CK) on follow-up testing. Given that this rise is mild and has occurred in the context of clear clinical improvement, without any worsening of weakness or other features suggestive of active myositis, was not felt to represent ongoing muscle inflammation. The patient is a physically active welder and reported increased levels of physical activity since feeling better on treatment, both of which are likely contributing factors. Overall, the mild CK elevation was more likely related to increased muscle exertion rather than persistent or worsening myositis.

Diagnosis relies on deep tissue biopsy including fascia, which in initial stages can show oedema and inflammatory infiltrates with lymphocytes, plasma cells, histiocytes and eosinophils in the deep fascia and deep subcutaneous tissue. In late stages, fascia can be thickened and sclerotic with no or little inflammatory infiltrates (25, 27).

MRI increasingly supports diagnosis by demonstrating fascial inflammation (T2 hyperintensity) and thickening in subcutaneous tissue and deep fascia (10, 12). Other imaging modalities such as 18F-fluorodeoxyglucose positron emission tomography/computed tomography (FDG-PET/CT) or ultrasound can be done if MRI is contraindicated or not feasible in cases where biopsy is inconclusive (29).

First-line therapy remains systemic corticosteroids, which induce remission in most patients (29–31). Prednisone is typically initiated at a dose of approximately 1 mg/kg/day following diagnosis, with gradual tapering over the subsequent 3–6 months based on clinical response (19). Prophylaxis against *Pneumocystis jirovecii* infection and bone protection should be considered to reduce treatment-related adverse effects. Eosinophilia often resolves rapidly, however, improvement in skin induration typically occurs more gradually over weeks to months.

Steroid-sparing immunosuppressive therapies, including methotrexate and mycophenolate mofetil, have been shown to improve outcomes and reduce the risk of relapse (19, 23, 31, 32). In refractory cases, biologic agents such as tocilizumab (33), rituximab (34), and infliximab (35), as well as Janus kinase inhibitors (36), intravenous immunoglobulin (37), and phototherapy (UVA-1 and psoralen plus UVA [PUVA]) (38), have demonstrated benefit in recent reports. Emerging therapies, including anti-interleukin-5 (anti-IL-5) agents, have also shown promise in selected cases and remain under investigation (39).

Physical therapy and early mobilization are essential to prevent joint contractures (40). In severe or refractory cases, surgical interventions may be required, including procedures for established contractures, fasciectomy, or carpal tunnel release, particularly in patients who do not respond adequately to conventional medical therapy (41).

Outcomes are generally favorable when treatment is initiated early; however, delayed recognition can lead to fixed fibrosis and functional disability (41). Long-term follow-up is advised due to risk of relapse and rare but serious hematologic complications such as aplastic anemia (19).

## Conclusion

Eosinophilic fasciitis can closely mimic systemic sclerosis and inflammatory myopathies. Making careful clinicopathological correlation essential to avoid misdiagnosis especially when patients may have positive autoantibodies such as PM-Scl-100. MRI and muscle biopsy are valuable diagnostic tools, as they demonstrate fascial inflammation and subcutaneous oedema and help distinguish fasciitis from primary myositis. Early recognition and prompt initiation of immunosuppressive therapy, including corticosteroids and steroid-sparing agents such as methotrexate, can result in significant clinical improvement and help prevent long-term morbidity.

## Patient perspective

Initial indications of something being wrong occurred when I couldn't bend my left leg at the knee fully back; and within a few weeks my left knee had become noticeably swollen. Additionally, I noticed that the inside of my left thigh had become very hard when touched. I experienced acute pain from my foot up to my thigh.

By March, I began feeling aches and pains in both legs. Within a few weeks, the pain escalated and became much more severe. To add to this, I began to experience a considerable amount of pain in both feet and it began to travel up my left leg, accompanied with a very tight feeling in my left calf and up into my left thigh. Subsequently, I was experiencing a lot of pain in my knee and a lot of stiffness in this area.

By June, the pain that I was experiencing could occur in one or both legs at any given time.

I noticed considerable skin discolouration surrounding my left knee whereby it changed to a mottled, brown color, and it was during this phase that I also began to notice that I was losing the hair on the inside of my left leg from the knee area upwards. Additionally, I noticed patches of whitened skin on my right abdomen and upper thigh which were unexplained. Furthermore, my left outer ankle area became incredibly swollen to the point that it had the appearance of a golf ball-sized lump protruding outwards. Each evening when I returned home from work, I could clearly see that my left lower leg, above the ankle, was extremely swollen from the sock line upwards.

When the pain and tightness were at their worst, the only thing that helped relieve the pain and pressure was wearing a knee brace. By wearing this, the pain and pressure were reduced to a more tolerable level; however, once I removed it, the pain, swelling, stiffness, and pressure returned within minutes.

I attended physiotherapy which, unfortunately, was completely ineffective.

From this time onwards, I developed a lot of pain and stiffness, more so first thing in the morning upon waking. I would wake up with immense pain and stiffness and it subsequently became extremely difficult for me to walk upon rising from bed. I struggled to hobble out of bed and had to withstand a lot of severe pain, attempting to walk in order to free out my leg. This excruciating pain and stiffness became worse as each week went by until I began my medication in August. Once I began medication, the pain and tightness began to dissipate gradually.

It is now December, and I'm feeling much better overall as my symptoms have reduced and become more manageable. I am still taking medication daily and will be for some time. I have a lot more good days than bad at present; I would estimate that the pain and tightness are at a lower level about 80% of the time, however, I still experience some bad days where the pain and tightness have returned at a much higher level. The tightness is now contained to my left calf and remains there constantly.

## Data Availability

The original contributions presented in the study are included in the article/supplementary material, further inquiries can be directed to the corresponding author.
